# Comparative Analysis of the Different Dyes' Potential to Assess Human Normal and Cancer Cell Viability *In Vitro* under Different *D*/*H* Ratios in a Culture Medium

**DOI:** 10.1155/2020/2373021

**Published:** 2020-02-25

**Authors:** I. A. Zlatskiy, A. V. Zlatska, N. V. Antipova, S. A. Dolenko, I. M. Gordiienko, O. S. Gubar, R. G. Vasyliev, D. A. Zubov, S. N. Novikova, A. V. Syroeshkin

**Affiliations:** ^1^Peoples Friendship University of Russia (RUDN University), 6 Miklukho-Maklaya St, Moscow 117198, Russia; ^2^State Institute of Genetic and Regenerative Medicine NAMS of Ukraine, 67 Vyshgorodska Str., Kyiv 04114, Ukraine; ^3^Biotechnology Laboratory Ilaya Regeneration, Medical Company Ilaya, Kiev, Ukraine; ^4^Shemyakin-Ovchinnikov Institute of Bioorganic Chemistry, RAS, Moscow, Russia; ^5^Institute of Colloid and Water Chemistry, National Academy of Sciences of Ukraine, Kyiv 03680, Ukraine; ^6^RE Kavetsky Institute of Experimental Pathology, Oncology and Radiobiology, National Academy of Sciences of Ukraine, Kyiv, Ukraine

## Abstract

In this study, using new approach (laser diffraction + biological dyes), we have demonstrated the decrease of cells viability *in vitro* in the deuterated growth medium, whereas in the deuterium-depleted medium, there was an increase of cell viability. We have also found that not all dyes are equally sensitive to the *D*/*H* ratios in the culture medium (system) as well as to the different cell types (cancer vs normal cells).

## 1. Introduction

In modern cell biology and cytology, it is often required to evaluate a set of parameters characterizing the viability of various cell population types [1, 2]. Determining cell viability is necessary to assess their morphological integrity, for example, after the cell line is removed from cryopreservation, as well as to study the effect of various stimuli (different cultivation conditions, the impact of toxins, pharmacological drugs, various pathogenic factors, etc.) [3–5].

Cell viability is defined as the ability of cells to perform specific functions, as well as the ability to realize the mitotic potential of cells capable of division [6]. There are several different methods to assess the viability of a cell culture or tissue sample, depending on the specific task of the study [7, 8]. They can be divided into the following groups: assessment of the cell morphological integrity (nonspecific assessment of viability) and assessment of the cell metabolic activity [9, 10].

Methods of nonspecific assessment of the cell culture viability suggest visual discrimination between viable and dead cells after staining with specific dyes which penetrate into the cell only if its membrane is damaged. Thus, stained cells are recognized as dead. The staining results are evaluated either by microscopy and counting of the proportion of dead cells relative to their total number, or by flow cytofluorimetry. The most common dyes routinely used in cell cultivation to this end are trypan blue and propidium iodide (PI).

More sophisticated and accurate methods for assessing viability include determining the ability of cells to perform their functions [11]. One of the ways is to assess the level of specific substances that are produced by cells in a normal state [12]. The decrease in the concentration of these substances indicates that the externally intact cell may be functionally defective, that is, not viable (metabolic activity) [13].

In our study, we used several groups of biological dyes with different chemical and physical parameters to evaluate their potential to assess the viability of cell cultures. As a factor that can significantly affect the viability of cultures, we used the deuterium/protium ratio in the culture media of normal and cancer cell lines *in vitro*. Deuterium (*D*) in aqueous solutions can stimulate or inhibit metabolic processes in living systems [14–16]. Studies of physical and chemical properties of water with different hydrogen isotopes ratio revealed abnormal phenomena in water with either reduced or increased deuterium content, associated with a huge isotopic effect [17–19].

Thus, the aim of our study was to investigate the effectiveness of different dyes to assess the viability of numerous cell culture types *in vitro* under different deuterium content conditions.

## 2. Materials and Methods

### 2.1. Substances and Solutions under Investigation

Some structural, physical, and chemical features of test substances (dyes) are presented in Table 1.

Dyes used for viability assessment are FDA (fluorescein diacetate) (Sigma-Aldrich, USA); PI (propidium iodide) (Sigma-Aldrich, USA); and trypan blue (Sigma-Aldrich, USA).

The dye used for metabolic activity assessment is Alamar blue (redox indicator) (Thermo Fisher, USA).

Growth media on water with different deuterium contents (DDW and D_2_O) were used (Sigma-Aldrich, USA).

### 2.2. Physicochemical Analysis of Water with Different Deuterium Contents

The following basic water samples with different deuterium contents were used in this study: deuterium-depleted water (DDW) with D/H = 4 ± 2 ppm (Sigma-Aldrich, USA) and D_2_O water with *D*/*H* = 99 absolute at % *D* (Sigma-Aldrich, USA). Growth media with various deuterium contents were prepared by diluting deuterium-depleted and deuterated water. The following growth media were used in this study: (1) DDW medium: medium with *D*/*H* ratio 35 ppm; (2) D_2_O medium: the medium with the highest deuterium contents, i.e., *D*/*H* ratio 500.000 ppm. The Milli-Q water served as a standard (Control medium) with *D*/*H* ratio 150 ppm. Milli-Q, deuterium-depleted, and deuterated water had no differences in physical characteristics [20] or in trace element composition, except the deuterium content. This excluded the multifactor influence in the system for all comparison groups. The deuterium/protium content was controlled by multipass laser absorption spectroscopy on the Isotopic Water Analyzer-912-0032 (Los Gatos Research Inc., USA), according to the manufacturer's recommendations. Detailed description of this method was presented in previous studies [14].

Chemical analysis of water with different deuterium contents was performed by inductively coupled plasma-mass spectrometry on the ICP-QMS Agilent 7500CE spectrometer (Agilent Technologies, USA) [21]. Calibration solutions with a high range of elements' concentration (from 0.1 *μ*g/dm^3^ to 100 *μ*g/dm^3^) were used for device calibration. The solutions were prepared based on the international standard 2.74473.0100 “ICP Multi-Element Standard Solution XXI CertiPUR®” which contains the following elements: Ag, Al, As, Ba, Be, Bi, Ca, Cd, Co, Cr, Cs, Cu, Fe, Ga, In, K, Li, Mg, Mn, Na, Ni, Pb, Rb, Se, Sr, Tl, U, V, Zn, and Hg. The concentration of all above-listed 24 elements in the Milli-Q, deuterium-depleted, or heavy water did not exceed the upper detection limit (detection limit range: 0.1–10 ppm). Detailed description of the methods was presented in previous studies [14, 22].

### 2.3. Physical and Chemical Parameters in Growth Media with Different Deuterium Contents

pH, redox potential (RP), electrical conductivity (EC), and salinity were controlled with an 8200 pH/ORP/conductivity/TDS/Salt/temp. meter (EZODO, Taiwan).

### 2.4. Laser Diffraction Method for Assessing Cell Culture Metabolic Activity

Particle size analysis (numerical and volume distribution of particle size/dimension spectra) and dissolution kinetics of powders of different dispersity ranges were recorded by the method of low-angle laser light scattering (LALLS) on diffraction particle analyzers [23] using Master Sizer 2000 instrument, Zeta Sizer Nano ZS instrument (MALVERN Instruments, UK), and “Cluster-1,” IDL-1, laser meter of dispersion (manufactured by the Institute of Colloid Chemistry and Chemistry of Water, Ukraine; modification RUDN, Russia). Detailed description of the methods was presented in previous study [24, 25].

### 2.5. Documents for Working with Cell Cultures In Vitro

The experiments with human cell cultures were carried out in accordance with the human experiment issues of the Code of Ethics of the World Medical Association (Declaration of Helsinki). In all cases, donors signed the voluntary informed consent forms.

The cells were cultured using standard protocols [26, 27]. In the experiment with the normal human cell culture (fibroblasts and adipose derived stem cells) *in vitro*, materials from donors were used.

### 2.6. Culturing of Normal Cell Lines of Adipose-Derived Stem Cells and Human Dermal Fibroblasts In Vitro

Detailed description of the methods was presented in previous study [28]. Human dermal fibroblasts (hDFB) were isolated from the skin biopsy samples of the skin biopsy samples. Human adipose-derived stem cells (hADSCs) were isolated from the lipoaspirate. The skin samples and the lipoaspirate were incubated in 0.1% collagenase IA and 0.1% pronase with the addition of 2% FBS in modified MEM;-*α* (all from Sigma, USA) for 2 hours at +37°C. The resulting cell suspension was washed twice with PBS (Sigma, USA) and was transferred to a 25 cm^2^ cell culture flask (SPL, Korea). Cells were cultured in the following control growth medium: modified MEM;-*α* (Sigma-Aldrich, USA) prepared from the powder by dilution with Milli-Q water of natural isotope content supplemented with 10% FBS (Sigma-Aldrich, USA), 2 mM L-glutamine, 100 U/ml penicillin, 100 *μ*g/ml streptomycin, and 1 ng/ml FGF-2 (all from Sigma-Aldrich, USA). Experimental growth media had a composition similar to the control one but were prepared on the basis of deuterated and deuterium-depleted waters. The cells were cultured in a multigas incubator CB210 (Binder, Germany) at +37°C in the atmosphere of saturated humidity, 5% CO_2_, and 5% O_2_.

Cells were seeded with a density of 1000 cells per 1 cm^2^. In 24 h, the control growth medium was changed for the experimental one.

To determine the effect of deuterium on the hADSC and DFB biological properties, cell cultures from five different donors were assessed at P2, after 5 days of culturing in experimental media.

### 2.7. Culturing of Cancer Cell Lines *In Vitro*

Detailed description of the methods was presented in previous study [22]. Human cancer cell lines A549 (lung carcinoma), HT29 (colon adenocarcinoma), and U87 (glioblastoma) were cultured in DMEM prepared from the powder by dilution in the DDW or Milli-Q water with natural isotopic content and sterilized by filtering. The media are further referred to as DDW medium and control medium. The culture media were additionally supplemented with 10% FBS and 2 mM glutamine (all from Sigma, USA). The cells were cultured in CO_2_ incubators CB210 (Binder, Germany) at +37°C in the atmosphere of absolute humidity and 5% CO_2_.

A549, HT29, and U87 cell lines were seeded in 24-well or 6-well plates at a density to reach 90% confluence in 24 hours. Afterwards, the cells were cultured in serum-free medium (to inhibit cell proliferation). Then, the cells were washed three times and the culture medium was completely changed for the experimental medium for each group.

To determine the effect of deuterium on the biological properties of A549, HT29, and U87 cell lines, three cell lines at P2 were assessed, after 6 days of culturing in experimental media.

In the experiment with cancer human cell lines *in vitro* (A549 (lung carcinoma), HT29 (colon adenocarcinoma), and U87 (glioblastoma)), material from American Type Culture Collection (ATCC) was used.

### 2.8. FDA and PI Assay for Cell Viability Assessment

Standard protocol was used for the FDA and PI assay [29]. The cells were stained with РI (propidium iodide) (Sigma-Aldrich, USA) and FDA (fluorescein diacetate) (Sigma-Aldrich, USA) after 24 h and 72 h in culture. PI does not penetrate into living cells; therefore, it stains only those in which the integrity of the cell membrane is broken. FDA is not a fluorescent molecule, but when penetrated into living cells, it is converted into a fluorescent form, fluorescein. The number of dead and living cells in different groups was counted using fluorescence microscopy (FITC and Texas Red filters; Carl Zeiss, Germany).

Viability was calculated as the ratio of living cells to the total number of cells and was expressed as a percentage according to the formula:

(1) $$\begin{array}{c} \text{cell viability}=\left(\frac{\text{number of living cells}}{\text{total number of cells}}\right)\times 100\%. \end{array}$$

### 2.9. Alamar Blue Assay for Cell Viability Assessment

A standard protocol was used for Alamar Blue assay [30]. In brief, 10% of Alamar Blue (redox indicator; Thermo Fisher, USA) was added to the culture medium at 24 h and 72 h and incubated for 3 h [31]. Reduced Alamar Blue was detected at 540 nm vs 630 nm using a Labsystems Multiskan PLUS spectrofluorimeter (USA). Cell metabolic activity was calculated according to the following formula:

(2) $$\begin{array}{c} \%\text{ of reduction}=\frac{\left(\left(\varepsilon ox\right)\lambda 2\cdot A\lambda 1-\left(\varepsilon ox\right)\lambda 1\cdot A\lambda 2\right)\text{ experiment}}{\left(\left(\varepsilon ox\right)\lambda 2\cdot A^{\prime }\lambda 1-\left(\varepsilon ox\right)\lambda 1\cdot A^{\prime }\lambda 2\right)\text{ control}}, \end{array}$$

where *λ*1 = 540 nm, *λ*2 = 630 nm, (εox) *λ*2 = 34,798, (*εox*) *λ*1 = 47,619, *Aλ*1 is the experimental sample absorption at *λ*1 = 540 nm, *Aλ*2 is the experimental sample absorption at *λ*2 = 630 nm, *A*′ *λ*1 is the control sample absorption at *λ*1 = 540 nm, and *A*′ *λ*2 is the control sample absorption at *λ*1 = 630 nm.

### 2.10. Trypan Blue Assay for Cell Viability Assessment

A standard protocol was used for Trypan blue assay [32]. In brief, 0.2–0.4% phosphate-saline buffer solution of the dye was introduced into the experimental medium. Cells were incubated with a dye for 10 minutes at 37°C and counted in a Countess™ II Automated Cell Counter (Thermo Fisher, USA). The optimal cell number range (1 × 10*e*5 to 4 × 10*e*6 cells/mL) was used.

The measurements were carried out at 24 and 48 hours of the experiment in normal and cancer cell cultures, in culture media based on DDW, D_2_O, and the control medium.

### 2.11. Statistics

All statistical data processing was performed using Student's *t*-test in Origin Pro software. Differences between the comparison groups were considered reliable at *p* < 0.05, and values are expressed as mean ± SD. The viability in the control group was set at 100%, and in the comparison groups, the changes are given as percentage of control.

## 3. Results and Discussion

### 3.1. Physical and Chemical Parameters of Growth Media with Different Deuterium Contents

We found significant differences in physicochemical parameters (рH, RP, EC, and salinity) in different comparison groups before and after cultivation of hADSCs in media with different *D*/*H* ratios (Table 2). Before cultivation, all groups had the same performance. After cultivation, a decrease in pH and an increase in RP were observed in all the groups.

### 3.2. Particle Size Analysis for Assessing the Cell Culture Metabolic Activity in Growth Media with Different Deuterium Contents

**Figure 1:**
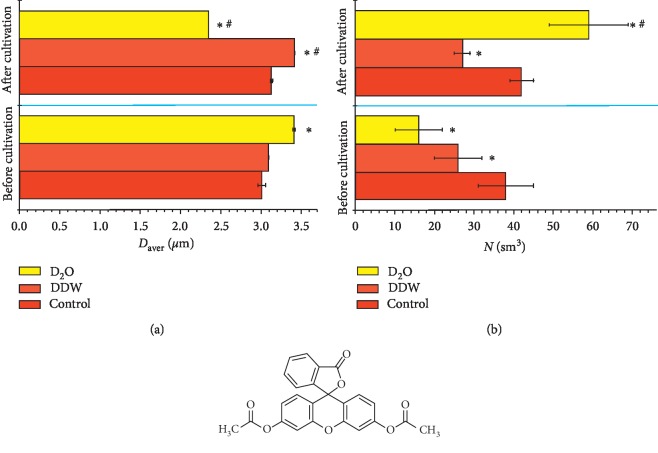
Change of size (*D*_aver_) (a) and number *N* (b) of optical inhomogeneities in media with different *D*/*H* ratios before and after the ADSC cultivation *in vitro*. (*Т* = 12°С). The results are expressed as mean ± SD (*n* = 6); ^*∗*^*p* < 0.05 compared to the control group; ^#^*p* < 0.05 compared to the same medium before cultivation.

Using the laser diffraction method, we have found essential differences in structural inhomogeneities in culture media with different *D*/*H* ratios (Figure 1). This suggests that various groups of molecular and supramolecular structures were formed in the experimental systems.

Before cultivation, the smallest size and, accordingly, the largest number of optical inhomogeneities were observed in the control sample. It should be noted that the DDW medium was slightly different from the control medium. D_2_O medium was characterized by larger size and, accordingly, a smaller number of optical inhomogeneities (Figure 1).

After cultivation, the following tendency was observed: an increase in the deuterium content in a sample led to a noticeable decrease in the size of optical inhomogeneities and, accordingly, to an increase in their number. In this case, there was a multidirectional influence of the deuterium content compared to the corresponding medium before cultivation. So, there was a slight increase in the size of optical inhomogeneities in low deuterium concentrations (DDW medium), and, on the contrary, a decrease in a sample with high deuterium content (Figure 1(a)). The reverse trend is observed for the number of optical inhomogeneities (Figure 1(b)). In the control medium, we did not observe changes before and after cultivation.

Changes in physicochemical parameters (Table 2) and structural optical inhomogeneities (Figure 1) can be explained by several factors. The deuterium/protium ratio can directly change the supramolecular structure of the medium, without influencing intracellular metabolism, as was noted earlier for the water with low salt composition [14, 22]. However, it is likely that different *D*/*H* ratios may cause different effects associated with the cell culture metabolic activity. Probably, in the studied media, cells secrete metabolites of a different nature, respectively, and various structural heterogeneities are formed, which are detected using laser diffraction. Thus, changes in inhomogeneities in culture media could be the result of changes in intracellular metabolism caused by different *D*/*H* ratios.

Considering the different metabolism of cells in culture media with different *D*/*H* ratios, we decided to compare different groups of biological dyes whether they have the same potential as quantitative viability indicators for our experimental system.

### 3.3. Cell Viability Assessment with Alamar Blue Dye after Culturing in Growth Media with Different Deuterium Contents

Table 3 represents results of the Alamar blue assay assessment of the normal and cancer cell metabolic activity *in vitro* in culture media with different *D*/*H* ratios at 24 h and 72 h. The Alamar blue test system is based on the resazurin reduction to resorufin by metabolically active living cells. This is an intracellular conversion performed by mitochondrial, microsomal, and cytosolic oxidoreductases. Thus, Alamar blue presents an integrative index of cellular metabolic activity which is determined by plasma membrane integrity, glycolysis intensity, mitochondria respiratory chain, and synthesis processes activity [30, 31, 33].

Our study showed that the most intensive metabolic activity was observed in cells cultivated in growth medium with DDW, whereas the growth medium with high deuterium concentration inhibited metabolic activity (Table 3). There was a reliable metabolic activity decrease at 24 h and 72 h in hADSCs, A549, and HT29 cultures in the D_2_O medium, which could evidence the deuterium cumulative effect and chronic toxicity. A reliable increase in hADSCs and hDFB metabolic activities was observed at 24 h and 72 h in the DDW medium. Metabolic activities of U87, A549, and HT29 cancer cell lines in the DDW medium remained the same as in the control medium.

### 3.4. Cell Viability Assessment with FDA and PI Dyes after Cultivation in Growth Media with Different Deuterium Contents

Table 4 represents results of FDA and PI staining in order to assess the normal and cancer cell viability in culture media with different *D*/*H* ratios at 24 h and 72 h. PI does not penetrate into living cells; it stains only dead cells in which the integrity of the cell membrane is broken. FDA is not a fluorescent molecule, but when penetrated into living cells, it is converted into a fluorescent form, fluorescein.

The highest cell viability *in vitro* was observed in the control medium with a natural *D*/*H* ratio. The cell cultures in the DDW medium demonstrated similar viability rate, except for the hADSCs, where a reliable decrease in viability was observed at 72 h. Significant deuterium toxicity was detected in the most deuterated growth medium in hADSCs and hDFB cultures. At the same time, the U87 cancer line viability after the cultivation in the D_2_O medium was not below control. Therefore, the determination of viability by FDA and PI staining may not always be reliable for different cell types (normal/cancer) *in vitro*.

### 3.5. Cell Viability Assessment with Trypan Blue Dye after Cultivation in Growth Media with Different Deuterium Contents

Table 5 represents results of trypan blue staining in order to assess the normal and cancer cell viability in culture media with different *D*/*H* ratios at 24 h and 72 h. Trypan blue is an acidic aniline dye used for selective staining of dead cells and tissues. This dye is not able to penetrate the cell through the intact cell membrane, so living cells are not stained. If the membrane is damaged, trypan blue stains the cell nucleus in an intensely blue color. Stained cells are considered dead.

We have found no significant differences between comparison groups. Therefore, it seems that the use of trypan blue is not effective for cell viability assessment under the conditions of the modified *D*/*H* ratio.

Previously, it was shown that either deficiency or excess of deuterium could be negative for cell activity *in vitro* [33, 34]. It should be noted that the study of deuterium as a component of living systems is not limited only by its biological role evaluation [15]. There is a theory of density inhomogeneities in water with different deuterium contents, which suggests that deuterium, depending on its concentration, acts as a control element of water's physicochemical properties [14]. According to this theory, exchange processes, diffusion, etc., have higher reaction kinetics in deuterium-depleted water [17]. Our data are in line with this theory as in the absence of density inhomogeneities in deuterium-depleted water, cell metabolic activity is intensified. Conversely, in deuterated water, all processes are slowed down that could be caused not only by the direct deuterium impact on the cell but also by increasing the nonuniformity of water density, which slows down metabolic processes in the extracellular environment [35, 36].

We should also consider the adaptation of cell cultures to the changed deuterium isotope composition in the growth medium. As demonstrated by the viability and metabolic activity assays, an increase in the indicator values is observed at 24 h in experimental groups followed by a decrease at 72 h. This could be considered as the so-called shock (functional) effect [28, 36–39] when the culture is transferred from one growth medium to another. However, redistribution of deuterium and protium in intracellular structures probably leads to a gradual adaptation of the culture to the new D/H isotope ratio. Over time span, this is reflected by an initial decrease in viability, followed by an increase in metabolic activity, which enables the proliferation of the culture *in vitro*.

Our data imply that, in fact, the widely used dyes for cell viability assessment are not equal or interchangeable and probably the variations in their action depend on the culture system tested. Thus, the tested dyes demonstrated discrepant results in our system with different *D*/*H* ratios in the culture medium. Some dyes (e.g., trypan blue) were not sensitive (i.e., we did not observe any difference between groups) and other dyes (e.g., FDA/PI) were only partly sensitive with respect to the cancerous or normal cell lines, whereas Alamar blue appeared the most sensitive and revealed differences between experimental groups both in cancer and normal cells. This can be explained by the fact that some features of deuterium/protium molecular interactions may contribute to the sensitivity of the viability assessment methods with different dyes. In particular, chemical interactions of dyes in deuterated or depleted in deuterium media may change the chirality of the active substances. On the other hand, we demonstrated the variability in the culture medium's physicochemical parameters depending on the *D*/*H* ratio and cell cultivation *in vitro* which could also lead to unpredictable changes in the dye sensitivity. However, these are only assumptions that require further research in this matter.

The study presents new data for determining the viability of cell culture *in vitro* using different groups of dyes under conditions of different deuterium contents in the medium. We have also used a new approach to assess cell viability using a combination of laser diffraction method and viability dye staining. The change in optical inhomogeneities measured by laser diffraction could serve as markers of metabolic changes which are further supplemented by viability dye staining. The combination of these methods could reveal the active substance impact on the cell culture (in our case, the *D*/*H* ratio in the culture medium). However, we believe that this approach (laser diffraction + viability dye) is not limited to our experimental system and can be used in assessing the effect of substances of different nature on cultures of normal and cancer cells *in vitro*, but precautions should be taken regarding the dye's nature and sensitivity in each particular case. Molecular materials, in which hydrogen-bonding frequently plays an important role, are particularly susceptible to isotopic substitution of hydrogen, leading to significant changes in structure, as well as electronic, magnetic, and vibrational alterations. Understanding and predicting these differences would be extremely useful for chemists and physicists working in this area. It would be particularly important in the biomedical sciences, where these considerations can lead to changes in the effect of a particular drug. In our opinion, studying the determination of cell viability under conditions of different ratios of deuterium/protium using the laser diffraction method will help to understand these issues.

## 4. Conclusion

We have revealed essential differences in the effectiveness of biological dyes for assessing the viability of cell cultures *in vitro* of cancer and normal lines using different *D*/*H* ratios in the culture medium as a model system. Our approach can serve as a basis for the selection of viability dyes for cytotoxicity evaluation of active substances of different nature, not limited to the *D*/*H* ratio.

## Figures and Tables

**Table 1:** Structure and physical and chemical features of test substances (dyes).

Structures and chemical formula	Names and identifiers	Molecular weight (g/mol)	Solubility in water
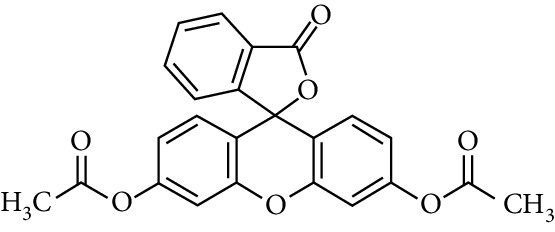 C_24_H_16_O_7_	Fluorescein diacetate, (6′-acetyloxy-3-oxospiro[2-benzofuran-1,9′-xanthene]-3′-yl) acetate	416.4	Slightly

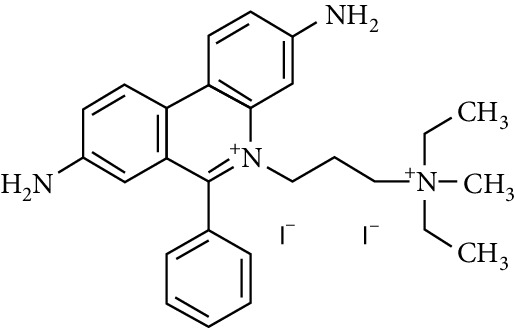 C27H34I2N4	Propidium iodide,3-(3,8-diamino-6-phenylphenanthridin-5-ium-5-yl)propyl-diethyl-methylazanium; diiodide	668.4	Slightly

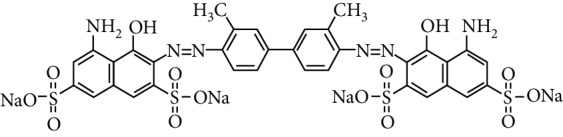 C34H24N6Na4O14S4	Trypan blue, tetrasodium; 5-amino-3-[[4-[4-[(8-amino-1-hydroxy-3,6-disulfonatonaphthalen-2-yl)diazenyl]-3-methylphenyl]-2-methylphenyl]diazenyl]-4-hydroxynaphthalene-2,7-disulfonate	960.8	Soluble

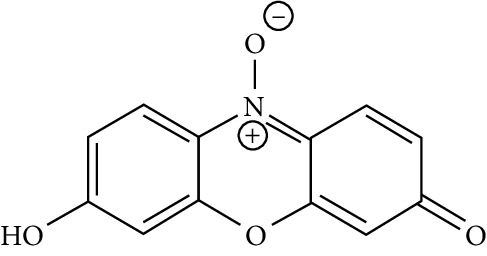 C12H7NO4	Resazurin, 7-hydroxy-10-oxidophenoxazin-10-ium-3-one	229.19	Soluble

**Table 2:** Physical and chemical parameters of growth media with different deuterium content before and after the hADSС cultivation *in vitro* (*Т* = 12°С).

Culture medium	Parameters			
	pH	RP	EC (*μ*S)	Salinity (ppm)
Control medium before cultivation	8.09	−60.1	3.13	2.05

Control medium after cultivation	7.22	−11.6	4.52	2.91

DDW medium before cultivation	7.89	−52.6	4.96	3.27

DDW medium after cultivation	7.13	−7.0	5.31	3.49

D_2_O medium before cultivation	7.90	−51.0	4.30	2.83

D_2_O medium after cultivation	6.96	+1.7	4.42	2.91

**Table 3:** Metabolic activity of human normal and cancer cells *in vitro* in culture media with different deuterium contents. Alamar blue assay was performed after 24 h and 72 h of cell cultivation in control or experimental growth media (the results are expressed as mean ± SD, *n* = 5, ^*∗*^*p* < 0.05 compared to the control group).

Exposure time (h)	24			72		
Culture medium (%)	Control	DDW	D_2_O	Control	DDW	D_2_O
Metabolic activity of А549 cell line	100 ± 3	108 ± 6	69 ± 7^*∗*^	100 ± 4	105 ± 5	69 ± 14^*∗*^
Metabolic activity of HT29 cell line	100 ± 5	105 ± 3	72 ± 6^*∗*^	100 ± 6	107 ± 7	65 ± 11^*∗*^
Metabolic activity of U87 cell line	100 ± 3	110 ± 5	—	100 ± 5	108 ± 11	—
Metabolic activity of hADSCs	100 ± 2	162 ± 11^*∗*^	81 ± 8^*∗*^	100 ± 7	132 ± 13^*∗*^	69 ± 12^*∗*^
Metabolic activity of hDFB	100 ± 5	121 ± 7^*∗*^	—	100 ± 4	115 ± 8^*∗*^	—

**Table 4:** Viability of human normal and cancer cells *in vitro* in culture media with different deuterium contents. FDA/PI assay was performed after 24 h and 72 h of cell cultivation in control or experimental growth media (the results are expressed as mean ± SD, *n* = 5, ^*∗*^*p* < 0.05 compared to the control group).

Exposure time (h)	24			72		
Culture medium (%)	Control	DDW	D_2_O	Control	DDW	D_2_O
Viability of U87 cell line	100 ± 5	94 ± 5	95 ± 7	100 ± 8	92 ± 7	91 ± 5
Viability of hADSCs	100 ± 11	96 ± 10	75 ± 13^*∗*^	100 ± 12	88 ± 7^*∗*^	82 ± 9^*∗*^
Viability of hDFB	100 ± 8	98 ± 6	83 ± 8^*∗*^	100 ± 9	94 ± 4	85 ± 5^*∗*^

**Table 5:** Viability of human normal and cancer cells *in vitro* in culture media with different deuterium content. Trypan blue staining was performed after 24 h and 72 h of cells cultivation in control or experimental growth media (the results are expressed as mean ± SD, *n* = 5, ^*∗*^*p* < 0.05 compared to the control group).

Exposure time (h)	24			48		
Culture medium (%)	Control	DDW	D_2_O	Control	DDW	D_2_O
Viability of А549 cell line	100 ± 5	108 ± 12	103 ± 7	100 ± 9	105 ± 8	107 ± 9
Viability of HT29 cell line	100 ± 3	105 ± 6	104 ± 9	100 ± 8	107 ± 5	106 ± 12
Viability of U87 cell line	100 ± 7	102 ± 4	—	100 ± 11	108 ± 7	—
Viability of hADSCs	100 ± 8	110 ± 10	108 ± 12	100 ± 6	102 ± 4	110 ± 11
Viability of hDFB	100 ± 11	109 ± 13	107 ± 12	100 ± 7	111 ± 9	104 ± 7

## Data Availability

The experimental data used to support the findings of this study are available from the corresponding author upon request.
